# piRNA and miRNA Can Suppress the Expression of Multiple Sclerosis Candidate Genes

**DOI:** 10.3390/nano13010022

**Published:** 2022-12-21

**Authors:** Saltanat Kamenova, Aksholpan Sharapkhanova, Aigul Akimniyazova, Karlygash Kuzhybayeva, Aida Kondybayeva, Aizhan Rakhmetullina, Anna Pyrkova, Anatoliy Ivashchenko

**Affiliations:** 1 Higher School of Medicine, Faculty of Medicine and Healthcare, Al-Farabi Kazakh National University, Almaty 050040, Kazakhstan; 2 Department of Nervous Diseases, Asfendiyarov Kazakh National Medical University, Almaty 050012, Kazakhstan; 3 Department of Technology of Production of Livestock Products, A. Baitursynov Kostanay Regional University, Kostanay 110000, Kazakhstan; 4 Institute of Biochemistry and Biophysics, Polish Academy of Sciences, 02-106 Warsaw, Poland; 5 Center for Bioinformatics and Nanomedicine, Almaty 050060, Kazakhstan

**Keywords:** multiple sclerosis, genes, miRNA, piRNA, diagnosis

## Abstract

Multiple sclerosis (MS) is a common inflammatory demyelinating disease with a high mortality rate. MS is caused by many candidate genes whose specific involvement has yet to be established. The aim of our study was to identify endogenous miRNAs and piRNAs involved in the regulation of MS candidate gene expression using bioinformatic methods. A program was used to quantify the interaction of miRNA and piRNA nucleotides with mRNA of the target genes. We used 7310 miRNAs from three databases and 40,000 piRNAs. The mRNAs of the candidate genes revealed miRNA binding sites (BSs), which were located separately or formed clusters of BSs with overlapping nucleotide sequences. The miRNAs from the studied databases were generally bound to mRNAs in different combinations, but miRNAs from only one database were bound to the mRNAs of some genes. For the first time, a direct interaction between the complete sequence of piRNA nucleotides and the nucleotides of their mRNA BSs of target genes was shown. One to several clusters of BSs of miRNA and piRNA were identified in the mRNA of *ADAM17, AHI1, CD226, EOMES, EVI5, IL12B, IL2RA, KIF21B, MGAT5, MLANA, SOX8, TNFRSF1A,* and *ZBTB46* MS candidate genes. These piRNAs form the expression regulation system of the MS candidate genes to coordinate the synthesis of their proteins. Based on these findings, associations of miRNAs, piRNAs, and candidate genes for MS diagnosis are recommended.

## 1. Introduction

The miRNAs (mRNA-inhibitory RNAs) regulate gene expression at the post-transcriptional level and play an important role in many cellular processes. Many genes whose expression depends on miRNA cause diseases, including multiple sclerosis (MS) [1,2,3,4,5]. A number of studies have suggested that multiple sclerosis can be diagnosed by using marker miRNAs as correlations between changes in miRNA concentrations and the disease have been found [6,7,8,9,10,11]. In recent years, the influence of small miRNAs on the development of MS has been actively studied [12,13,14,15,16,17]. Additionally, various methods of multiple sclerosis therapy involving the use of miRNAs have been proposed [18,19,20,21,22,23,24].

Considering that the number of candidate genes is in the several tens, and the number of miRNA is more than seven thousand, it is very difficult and expensive to determine in wet experiments which associations of miRNAs and target genes can be markers of MS. The use of computational technologies accelerates this task by a factor of thousands and significantly reduces the material costs of finding effective miRNA and candidate target gene associations. In general, elucidating the possible influence of miRNA on the expression of MS candidate genes is necessary, as there is only limited information on their relationships. We have used bioinformatic approaches to establish quantitative characteristics of the interaction between the miRNAs and mRNAs of candidate genes of various diseases, which have allowed us to identify how miRNAs are associated with candidate MS genes.

Note that most studies study only a few miRNAs whose expression correlates with MS disease and do not identify specific candidate target genes. For these reasons, there are many uncertainties in identifying effective associations between small miRNAs and candidate genes. This approach cannot adequately identify significant associations between miRNAs and candidate target genes, and from them, cannot select the most effective associations. One of the problems with elucidating the involvement of miRNAs in various diseases is the common misconception that miRNAs cause disease without the involvement of candidate genes. For example, correlations between changes in miRNA concentration and the development of disease are being established. In fact, miRNAs cause pathologies through their target genes. Moreover, some miRNAs regulate the expression of several or even hundreds of genes, and the expression of one gene depends on dozens of miRNAs [25]. Therefore, the common approach of detecting changes in a few miRNAs out of several thousand miRNAs through pathology without identifying their target genes out of more than 20 thousand human genes is highly inefficient. In this situation, only preliminary bioinformatic studies of the possible interactions between all miRNAs and mRNA candidate genes, as well as all human genes, can significantly and more objectively establish associations between the miRNAs and candidate genes involved in a particular disease. In the present study, we investigated the effects of miRNAs from the miRBase database (http://www.mirbase.org/ (accessed on 1 April 2022), and miRNAs from the studies of Londin et al. [26] and Backes et al. [27], to significantly increase the probability of detecting miRNAs involved in the development of MS.

We studied the possible effects of piRNA (PIWI-binding RNA) molecules on MS candidate genes. miRNAs are 20–25 nt and 5–9 nanometers long, whereas piRNAs are, on average, eight nucleotides longer than miRNAs (25–34 nt) [28] and, therefore, are nanoscale biological structures. The piRNAs can bind more strongly to the mRNA. Unfortunately, notions about the biological role of piRNAs have remained insufficiently substantiated over the many years since their discovery [29]. Some publications suggest that piRNAs can participate in the development of neurodegenerative diseases, but how this happens is unknown [30]. We hypothesized that piRNAs can bind to mRNAs and, as with miRNAs, suppress protein synthesis [31]. We tested this assumption in a recent study using the example of candidate genes involved in the development of multiple sclerosis. However, the putative mechanisms of this piRNA effect are highly questionable, and it is not even suggested that piRNA is involved in the regulation of candidate gene expression. At the same time, the interaction between piRNA and PIWI proteins involving the formation of complexes has been described. This suggests that the interaction between piRNAs and mRNAs is similar to the interaction between the RISC complexes of miRNAs and mRNAs. We are not aware of any attempt to determine the interaction between piRNAs and mRNAs by means of the known programs for miRNA–mRNA interaction determination, as the determination of the interaction between mRNAs and 26–35 nt piRNAs using the so-called “seed” programs is inadequate.

## 2. Materials and Methods

The nucleotide sequences of the RNAs candidate MS genes were downloaded from the NCBI website (http://www.ncbi.nlm.nih.gov (accessed on 1 April 2022)). The list of studied candidate MS genes is given in Table S1. The nucleotide sequences of the piRNAs were obtained from Wang et al. [28]. The 2567 miRNAs were taken from miRBase v.22 (http://www.mirbase.org (accessed on 1 April 2022)). The 3707 miRNAs were obtained from an article by Londin et al. [26], and the 1036 miRNA were obtained from an article by Backes et al. [27]. In order to establish the possible effect of miRNA and piRNAs on the MS candidate genes, we determined the interaction characteristics using the MirTarget program [32]. This program defines the following features of miRNA and piRNA binding to mRNA: (a) the initiation of the miRNA and piRNA binding to the mRNAs from the first nucleotide of the mRNAs; (b) the localization of the piRNA and miRNA BSs in the 5′UTR (5′-untranslated region), CDS (coding sequence), and 3′UTR (3′-untranslated region) of the mRNAs; (c) the schemes of nucleotide interactions between piRNAs, miRNAs, and mRNAs; (d) the free energy of the interaction between piRNA and the mRNA (ΔG, kJ/mol); (e) the ratio ΔG/ΔGm (%) is determined for each site (ΔGm equals the free energy of the piRNA binding with its fully complementary nucleotide sequence). The MirTarget program finds hydrogen bonds between adenine (A) and uracil (U), guanine (G) and cytosine (C), G and U, and A and C. Regarding the free energy of interactions (ΔG), a pair of G and C is equal to 6.37 kJ/mol, a pair of A and U is equal to 4.25 kJ/mol, and the pairs of G and U, and A and C are equal to 2.12 kJ/mol [33]. The distances between the bound A and C (1.04 nm), and G and U (1.02 nm) pairs are similar to those between the bound G and C, and A and U pairs, which are equal to 1.03 nm. The numbers of hydrogen bonds in the G–C, A–U, G–U, and A–C interactions were 3, 2, 1, and 1, respectively [34,35,36]. Consideration of the schemes shows which nucleotides of non-canonical pairs increase the energy of interaction between piRNAs and BSs.

The MirTarget program has proven itself useful in the search for associations of miRNAs and target genes in various diseases [37,38,39,40,41,42]. This program makes it possible to determine the quantitative characteristics of these miRNAs with mRNAs, which is very difficult to establish in wet experiments. Due to these characteristics, it is possible to assess the competition among miRNAs and piRNAs for binding to candidate target genes. The adequacy of the program in terms of finding BSs has been confirmed in several publications. A better confirmation of the obtained results as compared to “wet” experiments is provided by the schemes of interaction of nucleotides along the entire length of the miRNAs, piRNAs, and BSs. The schemes can be verified manually by finding the predicted piRNA BSs in the mRNA nucleotide sequence in the NCBI database.

## 3. Results

A list of MS target genes for miRNAs and piRNAs that indicates previous publications of the participation of the candidate genes in the development of MS is given in Table S1. Of these, *CD86, CD226, CLEC16A9, CYP27B1, FOXP3, IL2RA, IL-22RA2, IQGAP1,* and *MERTK* were targets for miRNAs, and *FCRL3, HLA-DRB1, MAPK1, MLANA, MYC, TALDO1,* and *TRIP11* genes were targets for piRNAs. The *ADAM17, AHI1, CD6, EOMES, EVI5, IL12B, KIF21B, MGAT5, SOX8, TAGAP, TBX21, TNFRSF1A, ZBTB46,* and *ZMIZ1* genes were targets for piRNA and miRNA.

### 3.1. miRNA Interactions with 5′UTR mRNA of MS Genes

The data presented in Table 1 indicate the presence of BSs for miRNAs with the 5’UTR of mRNAs of several candidate genes. Only single BSs were detected in the remaining mRNAs of six genes (Table S2). A specific feature of some MS candidate genes is the interaction of miRNAs groups with the mRNAs of two or more genes. For example, the 5’UTR mRNA of the *EVI5* gene contains a cluster of BSs of nine miRNAs that are 42 nt long. The sum of the lengths of these miRNAs BSs is 206 nt, which is 4.9 times the length of the cluster. This BS compaction results in a length-saving 5’UTR. However, we believe that the main purpose of the BSs compaction is to create competition between the miRNAs when binding to the mRNAs of the target gene to control its expression, resulting in more miRNAs binding with more free energy. Note that ID01702.3p-miR has three BSs, giving it an advantage in regulating *EVI5* gene expression. The mRNA of the *KIF21B* gene contains a 72 nt BSs cluster for nine miRNAs (Table 1), and thus, there is less competition between miRNAs because two miRNAs can bind in the cluster, such as ID03151.3p-miR and ID00049.5p-miR. The *SOX8* gene is less dependent on miRNAs because it has BSs in the 5’UTR mRNA for only four miRNAs. The *EVI5* and *KIF21B* genes have BSs for ID00296.3p-miR, ID01641.3p-miR, and ID01702.3p-miR in clusters. This indicates a relationship between the expression regulations of these genes. If the mRNA synthesis of the *EVI5* gene increases in the cell, these three miRNAs will bind to it more strongly, resulting in their lower inhibitory effect on the *KIF21B* gene mRNA. For MS diagnosis, the interaction between the free energy of miRNAs and the mRNA of the *EVI5* and *KIF21B* genes with a ΔG value of more than −130 kJ/mol is recommended. When selecting miRNA and target gene associations for diagnosis, the concentration of miRNAs should also be considered, as a high concentration of medium-interacting miRNAs can ultimately have a decisive effect on gene expression.

### 3.2. miRNA Interactions with CDS mRNA of MS Genes

The results of the miRNA interaction with CDS mRNA of MS candidate genes are provided in Table 2 and Table S3. A large BSs cluster was revealed in the CDS mRNA of candidate MS genes only for the *EOMES* gene (Table 2). For some miRNAs, the cluster had two to six BSs. Taking into account multiple BSs, the sum of the miRNAs lengths compared to the cluster length was 16.5 times greater. Associations between the *EOMES* gene and ID01702.3p-miR, ID02294.5p-miR, ID00296.3p-miR, ID01804.3p-miR, ID01041.5p-miR, ID01106.5p-miR, and ID02064.5p-miR are recommended for a diagnosis of disease. Note that the CDS mRNAs of the *EOMES* gene contains BSs for ID01702.3p-miR, ID00296.3p-miR, and ID02294.5p-miR, which bind to the 5’UTR of the mRNAs of the *EVI5* gene. In addition, the CDS mRNAs of the *EOMES* gene contained BSs for ID01702.3p-miR, ID00296.3p-miR, and ID00061.3p-miR, which bind to the BSs of the mRNAs cluster of the *KIF21B* gene. It was noted above that the *EVI5* and *KIF21B* genes have BSs for ID00296.3p-miR, ID02294.5p-miR, and ID01702.3p-miR in clusters, indicating a relationship between the regulation of expression of these genes (Table 1). The *EOMES* gene can also be added, whose expression depends on ID01702.3p-miR and ID00296.3p-miR. The BSs of the mRNA cluster of the *TNFRSF1A* gene contains predominantly miRNAs from the Backes database. This cluster has a high degree of compaction, as its length is 6.6 times shorter than the sum of the BSs of miRNAs.

### 3.3. miRNA Interactions with 3′UTR mRNA of MS Genes

The role of miRNAs from the Backes database (b-miRNA), which had many targets in the 3’UTR mRNA of the candidate MS genes, is surprising. Several MS candidate genes contained clusters of b-miRNA BSs in their mRNAs (Table 3 and Table S4). The 3’UTR mRNA of *ADAM17* contained a 33 nt long seven-miRNAs BSs cluster. Of these miRNAs, b-miR-1367-5p, b-miR-531-5p, b-miR-1641-5p, and b-miR-2038-5p had BSs in the mRNAs of the *AHI1* gene cluster (blue color). The mRNAs of the *ADAM17* and *AHI1* genes could complementarily bind with miR-619-5p and miR-5096, respectively.

The mRNA of the *CD226* gene contained four BSs clusters of predominantly b-miRNA (Table 3). The first BSs cluster from 5739 nt to 5763 nt could bind b-miR-1752-3p, b-miR-1441-3p, b-miR-1449-3p, b-miR-1189-3p, b-miR-1169-3p (completely complementary), miR-1273g-3p, and b-miR-2289-3p, and which could also bind to the fourth BSs cluster from 8642 nt to 8666 nt. This duplication of the BSs group of miRNAs indicates the importance of CD226 gene expression control by the identified miRNAs. In addition, b-miR-2083-3p and miR-5585-3p, as well as miR-1273g-3p and b-miR-2289-3p, each have two remote BSs. The b-miR-2038-3p has BSs in the mRNA of the *ADAM17*, *AHI1*, and *CD226* genes, and b-miR-2038-3p binds fully complementarily in the mRNA of the *CD226* gene.

The next target gene for many miRNAs was *EVI5*, in whose mRNA four large clusters of BSs, predominantly b-miRNAs, were identified (Table 3). The first cluster contained BSs for b-miR-1035-3p, b-miR-1752-3p, b-miR-1441-3p, b-miR-2164-3p, b-miR-1169-3p, b-miR-1189-3p, miR-1273g-3p, and b-miR-2289-3p, which bind in the *CD226* gene mRNA cluster. Some of these miRNAs (b-miR-1441-3p, b-miR-2164-3p, b-miR-1169-3p, b-miR-1189-3p, miR-1273g-3p, and b-miR-2289-3p) bind in the second cluster from 5498 nt to 5521 nt, and miR-1273g-3p binds completely complementarily.

In the last mRNA BSs cluster of the *EVI5* gene from 7373 nt to 7396 nt, the BSs of ID01836.5p-miR, b-miR-1367-5p, b-miR-1361-5p, b-miR-531-5p, b-miR-1131-5p, b-miR-1641-5p, b-miR-2038-5p, and b-miR-1608-5p were revealed. The BSs of these miRNAs were located in the mRNA of the *IL2RA* gene in an identical sequence (Table 3), and b-miR-531-5p, b-miR-1641-5p, b-miR-2038-5p, and b-miR-1608-5p bind completely complementarily.

**Figure 1:**
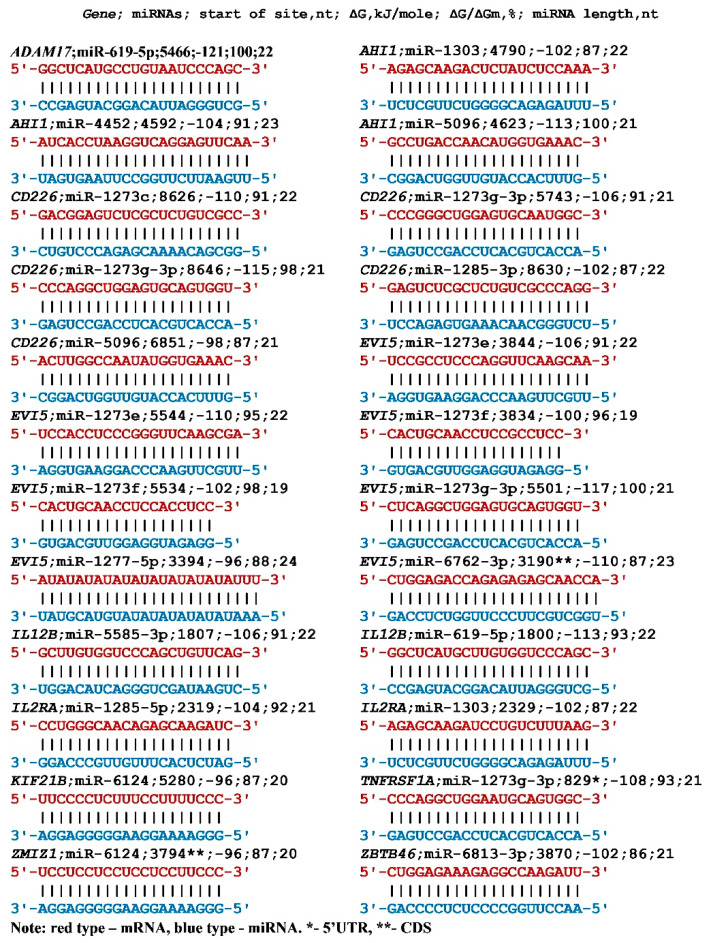
Schemes of miRNA (from miRBase) interaction with mRNAs of MS candidate genes.

The interaction of ID00436.3p-miR, ID01030.3p-miR, and miR-466 with the *MGAT5* gene mRNA is noteworthy (Table 3). The beginning of the BSs of these miRNAs are located two nucleotides apart and the BSs are repeated 13–14 times. Such multiple BSs greatly increase the binding probability of each miRNA, which increases the dependence of *MGAT5* gene expression on these miRNAs. In addition to a set of miRNAs organized into groups according to the principle of binding in clusters, miRNAs having one BS in the mRNA of the target gene can have a significant effect on the expression of candidate MS genes. Their effect depends on the ratio of the concentrations of miRNAs and mRNAs of the target gene. Quite often, readers have doubts about the reliability of the prediction of the BSs of miRNAs with mRNAs of target genes. To confirm the quantitative characteristics of the interaction between miRNAs and the mRNAs of target genes, we present schemes for the formation of hydrogen bonds between interacting canonical and non-canonical nucleotide pairs (Figure 1).

The given schemes convincingly demonstrate the interaction of nucleotides, and the values of the free energy of interaction are given. These characteristics were obtained based on semi-empirical values of the interaction of nucleotides in an aqueous medium due to hydrogen bonds, and in a comparative aspect, they reflect well these interactions of nucleotides between miRNA and mRNA target genes.

### 3.4. piRNA Interactions with 5′UTR mRNA of MS Genes

**Figure 2:**
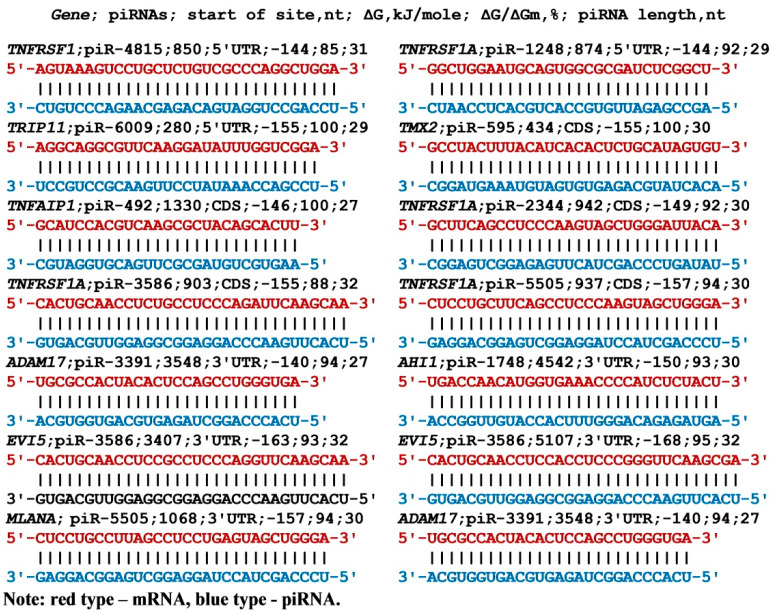
Schemes of interaction between piRNA and 5’UTR, CDS, and 3’UTR mRNA of MS candidate genes.

piRNAs can bind to the 5’UTRs of the mRNAs of candidate MC genes with a free energy higher than that of miRNAs (Table S5). When the piRNAs interact with mRNA candidate target genes, the free energy ranges from −140 kJ/mol to −159 kJ/mol. The mRNA of *TAGAP*, *TALDO1,* and *TBX21* genes each had a single BS. Expression of the MYC gene can be regulated by three piRNAs, and the ZBTB46 gene by six piRNAs. The BSs of the four piRNAs formed a cluster from 37 nt to 96 nt in the mRNA of the *ZBTB46* gene, resulting in competition between these piRNAs. *TNFRSF1A* gene expression could depend on eight piRNAs whose BSs formed a cluster 56 nt long, which was 6.4 times shorter than the sum of the piRNA lengths. Despite the considerable length of piRNA (27–34 nt), the interaction between the piRNA and mRNA nucleotides occurs along the entire length of the piRNA, which is clearly seen in Figure 2.

### 3.5. piRNA Interactions with CDS mRNA of MS Genes

The results of the piRNA effects on CDS mRNA candidate MS genes are shown in Table S6. Eight genes were targeted by one piRNA each. The mRNA of the *HLA-DRB1* gene contained two BSs, which were arranged with overlapping nucleotide sequences. The mRNAs of the *CD6* and *KIF21B* genes each contained three BSs located throughout the coding sequence. The mRNA of the *EOMES* gene could bind to five piRNAs, with the BSs of piR-5938, piR-5937, and piR-1344 forming a BSs cluster only 34 nt long. That is, the competition between these piRNAs for binding to mRNA was high.

The *TNFRSF1A* gene was targeted by 17 piRNAs whose BSs occupied a nearly continuous stretch of 102 nt. Given that the cluster of piRNA BSs in the CDS of the mRNA of the TNFRSF1A gene begins at 874 nt (Table 4) and continues into the CDS from 976 nt, the cluster of BSs is actually continuous and located in the CDS. This is the first time we have detected such a phenomenon. Enhanced control of *TNFRSF1A* gene expression by piRNA is apparently associated with its high risk of involvement in neurodegenerative diseases including multiple sclerosis.

### 3.6. piRNA Interactions with 3′UTR mRNA of MS Genes

The mRNAs of the *ADAM17* and *AHI1* genes have BSs for piR-16315, piR-5295, piR-5300, piR-5301, piR-5303, piR-5294, piR-6236, piR-7637, and piR-5358, which are part of mRNAs BSs clusters of both genes (Table 5).

Consequently, these piRNAs will compete to regulate the expression of both genes. The *ADAM17* and *AHI1* genes have different functions but are regulated by several of the same piRNAs involving other piRNAs. Hence, the regulation of the expression of these genes by such a set of piRNAs is necessary for the coordinated expression of these genes.

The cluster of nine piRNA BSs from 3501 nt to 3538 nt in the mRNA of the *ADAM17* gene is 37 nt long and is 7.3 times less than the sum of piRNA BSs. The mRNA of the *AHI1* gene can bind the same nine piRNAs that bind in the BSs cluster located from 4455 nt to 4491 nt. The total length of the piRNA BSs in this cluster is 271 nt and is 7.3 times the length of the cluster. A cluster of piR-5744, piR-7102, and piR-7105 BSs in the 3′UTR mRNA of the *AHI1* gene was detected in the 3′UTR mRNA of the *EVI5* gene at positions of 6872 nt, 6877 nt, and 6878 nt and in the 3′UTR mRNA of the *IL2RA* gene at positions 2115 nt, 2120 nt, and 2121 nt.

The most abundant in BSs piRNA clusters was the mRNA of the *EVI5* gene (Table 4). The first cluster of BSs consists of piR-10936, piR-10886, and piR-10885 BSs at positions 3378 nt, 3379 nt, and 3379 nt. These three piRNAs together with piR-1248, piR-10873, piR-11596, piR-10873, piR-10935, and piR-10934 form a BSs cluster from 5078 nt to 5108 nt in the 3′UTR mRNA of the *EVI5* gene and in the 3′UTR mRNA of the *MLANA* gene in positions from 1005 nt to 1035 nt.

A second BSs cluster of piR-4815, piR-10936, piR-10886, and piR-10885 in the 3′UTR mRNA of the *EVI5* gene was detected in the 5′UTR mRNA of the *TNFRSF1A* gene (Table 4). In the BSs cluster, piR-9994, piR-9059, piR-9036, piR3586, piR-7244, piR-5505, and piR-2344 bind from 3403 nt to 3475 nt. In the cluster of BSs from 5104 nt to 5139 nt, these same piRNAs can also bind in the 3′UTR mRNA of the *EVI5* gene.

A BSs cluster for piR-1254, piR-12623, piR-4112, and piR-12340 was located from 3517 nt to 3573 nt, and an identical BSs cluster from 4829 nt to 4886 nt was found in the 3′UTR mRNA of the *EVI5* gene. The BSs of piR-12346, piR-12399, piR-5505, piR-2344, piR-15405, piR-14239, and piR-15404 are located at a small interval after each of these clusters (Table 5).

The large BSs cluster for the ten piRNAs is located at 5215 nt in the 3′UTR mRNA of the EVI5 gene. The BSs of these same piR-1254, piR-12623, piR-17617, piR-11421, piR-12346, piR-12399, piR-15405, piR-14239, piR-15404, and piR-14625 BSs are located in the 3′UTR mRNA of the *MLANA* gene at 1142 nt.

Consequently, these piRNAs will compete with each other in two BSs clusters. The piR-12623 and piR-12399 BSs are located in the third mRNA site of the *EVI5* gene, which is also located at 39 nt. The results obtained indicate an increased dependence in EVI5 gene expression on many piRNAs. The schemes of interaction between piRNA nucleotides and 3’UTR mRNA nucleotides of the candidate genes clearly show a strong interaction between piRNA and mRNA (Figure 2).

Not all MS candidate genes were targeted by piRNA. The results of the characterization of piRNA interaction with the mRNA of the eight MS candidate genes are shown in Table 4. The piRNA BSs were predominantly located in the 3’UTR and only two genes were located in the 5’UTR and the *TNFRSF1A* gene in the CDS. The mRNAs of the *IL2RA*, *MGAT5*, and *ZBTB46* genes each had one piRNA BS, and the mRNA of the *MLANA* gene had three piRNA BSs. Each of the *ADAM17, AHI1, EVI5,* and *TNFRSF1A* genes was the target of several piRNAs whose BSs were located with overlapping nucleotides, which we called clusters of BSs. In the mRNA of the *ADAM17* gene, the cluster was located from 3508 nt to 3539 nt and was 8.4 times shorter than the sum of the BSs of the nine piRNAs. This compactization of BSs leads to competition between piRNAs when binding to the mRNA of the target gene.

As evidenced by the above piRNA and target gene interactions, the expression of genes that are targets of one or more of the same or different piRNAs is regulated in a linked manner. For example, an increase in the expression of a particular gene leads to the binding of the corresponding piRNA, which, in turn, will suppress less the expression of its other target genes. The decreased expression of a target gene of any piRNA will result in the suppression of other target genes of that piRNA. Consequently, piRNAs that have a set of target genes maintain a balance in the expression of their target genes.

Another aspect of the effect of piRNA and miRNA on a single gene is that at the beginning of ontogenesis, the proportion of synthesized piRNAs is greater than the proportion of miRNAs [36]. During phylogenesis, the piRNA fraction decreases and the miRNA fraction increases in differentiated cells. Therefore, piRNA and miRNA target genes will be less dependent on piRNA and more dependent on miRNA. The piRNA and miRNA target genes were *EOMES, ADAM17, AHI1, EVI5, IL2RA,* and *MGAT5*. These genes were most dependent on piRNA and miRNA, and therefore, their associations with the corresponding piRNA and miRNA are the most suitable for use in MS diagnosis.

## 4. Discussion

This work shows that to search targeting MS candidate genes for miRNAs, all known databases must be searched and used. If this requirement is ignored, it will be difficult to obtain unbiased data on the effect of miRNAs on candidate genes, including those of MS. The inclusion of piRNAs affecting MS candidate genes in the search significantly expands our understanding of the causes of MS development. It is known that piRNAs are synthesized predominantly in the early stages of ontogenesis and subsequently their synthesis continues in stem cells [43]. In contrast, miRNAs are weakly expressed in the initial stages of embryogenesis and are synthesized in most organs as the body tissues differentiate [43]. Note that in the present work, we found that some genes are simultaneously targeted by piRNA and miRNA. The *EOMES, ADAM17, AHI1, EVI5, IL2RA,* and *MGAT5* genes were targets for piRNA and miRNA (Supplementary Table S1). This reflects the different expressions of these genes at the initial stages of ontogenesis and during ontogenesis. These genes were most dependent on piRNA and miRNA, and therefore, their associations with the corresponding piRNA and miRNA are most suitable for use in MS diagnosis. If these genes are candidate disease genes, the likelihood of disease, particularly age-related disease, increases over the course of ontogeny. The identified interactions of several miRNAs and piRNAs in the mRNA of a gene, especially those interacting in clusters, necessitate monitoring the expression of candidate genes and piRNAs with miRNAs to reveal an objective assessment of the patient’s condition. The different interaction characteristics of different miRNAs and different piRNAs with the mRNA of the target gene require a comparison of their concentrations in combination with the expression of the target gene. As evidenced by the above piRNA and target gene interactions. The expression of genes that target one or more of the same or different piRNAs is regulated in a linked way. For example, an increase in the expression of a particular gene leads to the binding of the corresponding piRNA, which, in turn, will lead to less suppression of the expression of its other target genes. The decreased expression of a target gene of any piRNA will result in the suppression of other target genes of that piRNA. Consequently, piRNAs that have a set of target genes maintain a balance in the expression of their target genes. Another aspect of the effect of piRNA and miRNA on a single gene is that at the beginning of ontogenesis, the proportion of synthesized piRNAs is greater than the proportion of miRNAs. During phylogenesis, the piRNA fraction decreases, and the miRNA fraction increases in differentiated cells [43]. Therefore, piRNA and miRNA target genes will be less dependent on piRNA and more dependent on miRNA. The piRNAs and miRNAs have been shown to be key regulators of the expression of candidate MS genes. Establishing associations between piRNA, miRNA, and target MS candidate genes reveals how the expression of candidate genes depends on the concentrations of piRNA and miRNA. This will allow such associations to be used as markers of disease and for the development of subsequent therapies. Analysis of the interaction of miRNAs and piRNAs with the mRNA of MS candidate genes showed that there are groups of miRNAs and piRNAs that interact with the mRNA of one or two genes (these are colored in the tables). Measuring the concentration of such groups of miRNAs or piRNAs along with the expression of one or two candidate target genes provides a much better chance of determining which miRNAs or piRNAs regulate the expression of these genes and to what extent. Next, we need to determine which human genes are also targets of these miRNAs or piRNAs in order to establish what side-effects these miRNAs or piRNAs may have when used as diagnostic markers and as therapeutic agents.

## Figures and Tables

**Table 1:** Characteristics of miRNA interactions with 5′UTR mRNA of MS candidate genes.

Gene	miRNA	Start of Site, nt	ΔG, kJ/mol	ΔG/ΔGm, %	Length, nt
*EVI5*	ID00296.3p-miR	450	−142	91	25
	ID01641.3p-miR	450	−132	89	24
	ID01702.3p-miR	450 ÷ 460 (3)	−134 ÷ −138	89 ÷ 92	24
	ID01895.5p-miR	453	−132	89	24
	ID00756.3p-miR	454	−125	91	23
	ID02064.5p-miR	454, 458	−129, −132	90, 91	23
	ID02499.3p-miR	462	−121	93	21
	ID01595.3p-miR	470	−115	92	22
*KIF21B*	ID03151.3p-miR	195	−117	95	20
	ID00296.3p-miR	241	−138	88	25
	ID01641.3p-miR	241	−132	89	24
	ID01702.3p-miR	198	−134	89	24
	ID00061.3p-miR	204	−129	94	22
	ID01848.5p-miR	217	−117	89	23
	ID00049.5p-miR	227	−134	89	24
	b-miR-1045-5p	232	−119	90	23
	b-miR-1094-5p	245	−119	90	22
*SOX8*	ID02761.3p-miR	15	−134	90	24
	ID00278.3p-miR	19	−125	91	23
	ID01310.3p-miR	19	−121	92	22
	b-miR-1771-3p	19	−121	92	22

Note. In this and other tables, the number of miRNA BSs repeats is given in parentheses. Groups of miRNAs with BSs in different genes or the same gene are marked with the same color. The ÷ sign denotes the change in values in the “from” to “to” interval.

**Table 2:** Characteristics of miRNA interactions with CDS mRNA of MS candidate genes.

Gene	miRNA	Start of Site, nt	ΔG, kJ/mol	ΔG/ΔGm, %	Length, nt
*EOMES*	ID01702.3p-miR	759 ÷ 774 (3)	−134 ÷ −138	89 ÷ 92	24
	ID00061.3p-miR	761 ÷ 776 (6)	−125 ÷ −129	91 ÷ 94	22
	ID00296.3p-miR	767	−140	89	25
	ID02294.5p-miR	763 ÷ 769 (3)	−129 ÷ −136	88 ÷ 93	24
	ID00522.5p-miR	764	−127	91	23
	ID01041.5p-miR	770	−132	90	24
	ID00457.3p-miR	770	−127	94	22
	ID01873.3p-miR	770, 773	−123, −125	94, 95	21
	ID03151.3p-miR	770	−115	93	20
	ID01106.5p-miR	771	−132	89	24
	ID02064.5p-miR	772, 778	−132, −136	91, 94	23
	ID01879.5p-miR	772	−123	91	22
	miR-3960	772	−115	92	20
	ID02429.3p-miR	773	−125	92	23
	ID03367.5p-miR	773	−117	93	20
	ID01652.3p-miR	774	−125	89	23
	ID02538.3p-miR	774	−121	90	22
	ID02499.3p-miR	779	−119	92	21
	ID02368.3p-miR	782	−127	91	23
*TNFRSF1A*	b-miR-1752-3p	825	−110	96	22
	b-miR-1441-3p	826	−117	95	22
	b-miR-1449-3p	826	−106	93	22
	b-miR-2164-3p	827	−106	94	22
	b-miR-1189-3p	828	−106	94	22
	b-miR-1169-3p	828	−110	96	22
	miR-1273-3p	829	−108	93	22
	b-miR-2289-3p	830	−110	96	22

**Table 3:** Characteristics of miRNA interactions with 3′UTR mRNA of MS candidate genes.

Gene	miRNA	Start of Site, nt	ΔG, kJ/mole	ΔG/ΔGm, %	Length, nt
*ADAM17*	ID02997.5p-miR	5449	−113	93	22
	miR-619-5p	5466	−121	100	22
	b-miR-1367-5p	5510	−119	97	22
	b-miR-531-5p	5511	−104	96	19
	b-miR-1641-5p	5512	−98	96	18
	b-miR-2038-5p	5512	−98	96	18
	b-miR-1246-3p	5522	−104	91	21
	miR-1285-5p	5524	−104	92	21
*AHI1*	miR-619-5p	4546	−110	91	22
	b-miR-1620-3p	4561	−110	93	22
	miR-4452	4592	−104	91	23
	b-miR-754-5p	4621	−115	96	22
	miR-5096	4623	−113	100	21
	ID02175.3p-miR	4729	−113	93	22
	b-miR-1367-3p	4729	−115	89	23
	b-miR-2038-3p	4729	−117	92	23
	b-miR-2086-3p	4753	−123	89	25
	b-miR-1367-5p	4766	−110	90	22
	b-miR-531-5p	4767	−104	96	19
	b-miR-1641-5p	4768	−98	96	18
	b-miR-2038-5p	4768	−98	96	18
*CD226*	b-miR-1752-3p	5739	−104	91	21
	b-miR-1441-3p	5740	−110	90	22
	b-miR-1449-3p	5740	−113	98	21
	b-miR-1189-3p	5742	−104	92	20
	b-miR-1169-3p	5742	−108	94	20
	miR-1273g-3p	5743	−106	91	21
	b-miR-2289-3p	5744	−108	94	20
	b-miR-2087-3p	6952	−96	94	19
	b-miR-1608-3p	6964	−119	90	23
	ID02175.3p-miR	6966	−113	93	22
	b-miR-1367-3p	6966	−119	92	23
	b-miR-2038-3p	6966	−115	90	23
	b-miR-2086-3p	6990	−130	94	25
	ID01836.5p-miR	7002	−117	93	23
	b-miR-1361-5p	7003	−113	90	22
	b-miR-1367-5p	7003	−110	90	22
	b-miR-531-5p	7004	−102	94	19
	b-miR-1131-5p	7005	−98	94	19
	b-miR-1608-5p	7006	−102	94	19
	miR-1285-5p	7017	−108	96	21
	miR-1303	7027	−106	91	22
	miR−1273a	8624	−119	90	25
	ID01838.5p-miR	8625	−110	88	24
	miR-1273c	8626	−110	91	22
	b-miR-1246-5p	8631	−108	93	21
	b-miR-1035-3p	8634	−123	94	24
	b-miR-1752-3p	8642	−108	94	21
	b-miR-1441-3p	8643	−121	98	22
	b-miR-2164-3p	8644	−110	98	20
	b-miR-1189-3p	8645	−110	98	20
	b-miR-1169-3p	8645	−115	100	20
	miR-1273g-3p	8646	−115	98	21
	b-miR-2289-3p	8647	−110	96	20
	b-miR-1449-3p	8643	−110	96	21
	b-miR-2022-3p	8643	−106	93	20
	miR-5585-5p	8726	−110	95	22
	b-miR-2083-3p	8870	−106	100	20
	ID01334.3p-miR	8882	−113	90	22
*EVI5*	miR-1277-5p	3352	−100	92	24
	miR-1277-5p	3394	−96	88	24
	b-miR-1035-3p	3789	−117	89	24
	b-miR-1752-3p	3797	−106	93	21
	b-miR-1441-3p	3798	−119	97	22
	b-miR-2164-3p	3799	−108	96	20
	b-miR-1169-3p	3800	−106	93	20
	b-miR-1189-3p	3800	−108	96	20
	miR-1273g-3p	3801	−110	95	21
	b-miR-2289-3p	3802	−106	93	20
	miR-1273f	3834	−100	96	19
	b-miR-2164-5p	3834	−119	92	24
	b-miR-1927-5p	3838	−108	96	19
	miR-1273e	3844	−106	91	22
	b-miR-624-3p	4024	−98	96	20
	b-miR-1096-3p	3972	−102	94	20
	b-miR-2083-3p	4026	−102	96	20
	b-miR-1096-3p	5285	−100	92	20
	b-miR-1504-5p	5298	−119	87	25
	b-miR-609-5p	5304	−110	91	21
	b-miR-1441-3p	5498	−115	93	22
	b-miR-2164-3p	5499	−108	96	20
	b-miR-1169-3p	5500	−106	93	20
	b-miR-1189-3p	5500	−108	96	20
	miR-1273g-3p	5501	−117	100	21
	b-miR-2289-3p	5502	−106	93	20
	miR-1273f	5534	−102	98	19
	b-miR-2164-5p	5534	−125	97	24
	miR-1273d	5535	−121	89	25
	ID01404.5p-miR	5538	−113	91	23
	b-miR-1791-5p	5555	−102	100	19
	b-miR-1927-5p	5538	−108	96	19
	miR-1273e	5544	−110	95	22
	b-miR-624-3p	5586	−93	92	20
	b-miR-1096-3p	5670	−100	92	20
	b-miR-2131-3p	5681	−98	94	20
	ID01836.5p-miR	7373	−115	92	23
	b-miR-1367-5p	7374	−113	91	22
	b-miR-1361-5p	7374	−115	92	22
	b-miR-531-5p	7375	−104	96	19
	b-miR-1131-5p	7376	−102	98	19
	b-miR-1641-5p	7376	−98	96	18
	b-miR-2038-5p	7376	−98	96	18
	b-miR-1608-5p	7377	−104	96	19
	ID02199.5p-miR	7388	−113	90	23
*IL2RA*	ID01334.5p-miR	2066	−113	91	22
	miR-619-5p	2080	−110	91	22
	miR-5585-3p	2220	−106	91	22
	ID01836.5p-miR	2304	−115	92	23
	b-miR-1367-5p	2305	−117	95	22
	b-miR-1361-5p	2305	−119	95	22
	b-miR-531-5p	2306	−108	100	19
	b-miR-1131-5p	2307	−100	96	19
	b-miR-1641-5p	2307	−102	100	18
	b-miR-2038-5p	2307	−102	100	18
	b-miR-1608-5p	2308	−108	100	19
	miR-1285-5p	2319	−104	92	21
*MGAT5*	miR-107	3029	−110	91	23
	ID01261.5p-miR	4768	−110	93	20
	ID00436.3p-miR	4957 ÷ 4983 (14)	−104 ÷ −106	89 ÷ 91	23
	ID01030.3p-miR	4957 ÷ 4981 (13)	−108	89	23
	miR-466	4957 ÷ 4983 (14)	−106 ÷ −108	91 ÷ 93	23

**Table 4:** Characteristics of interaction between piRNA and CDS mRNA of MS genes.

Gene	miRNA	Start of Site, nt	ΔG, kJ/mol	ΔG/ΔGm, %	Length, nt
*TNFRSF1A*	piR-10936	874	−149	87	31
	piR-10886	875	−159	94	30
	piR-10885	875	−144	87	30
	piR-1248	874	−144	92	29
	piR-10873	875	−149	91	30
	piR-10935	875	−142	87	30
	piR-10934	875	−140	88	30
	piR-14091	882	−132	89	28
	piR-15670	882	−142	91	28
	piR-9994	900	−144	86	30
	piR-9059	900	−159	95	30
	piR-9036	901	−140	87	29
	piR-3586	903	−155	88	32
	piR-7244	906	−144	91	29
	piR-5505	937	−157	94	30
	piR-2344	942	−149	92	30
	piR-15406	946	−144	88	30

**Table 5:** Characteristics of interaction between piRNA and 3’UTR mRNA of MS genes.

Gene	miRNA	Start of Site, nt	ΔG, kJ/mol	ΔG/ΔGm, %	Length, nt
*ADAM17*	piR-16315	3501	−149	89	30
	piR-5295	3508	−146	85	31
	piR-5301	3509	−149	92	30
	piR-5300	3509	−144	92	30
	piR-5303	3509	−142	89	30
	piR-5294	3509	−140	85	30
	piR-6236	3509	−146	91	30
	piR-7637	3509	−153	95	30
	piR-5358	3509	−144	88	30
*AHI1*	piR-16315	4455	−144	86	30
	piR-5295	4461	−142	83	31
	piR-5301	4462	−149	92	30
	piR-5300	4462	−144	92	30
	piR-5303	4462	−142	89	30
	piR-5294	4462	−142	86	30
	piR-6236	4462	−146	91	30
	piR-7637	4462	−146	91	30
	piR-5358	4462	−151	92	30
	piR-6244	4494	−144	86	31
	piR-5744	4494	−144	87	31
	piR-7102	4500	−159	93	31
	piR-7105	4499	−153	86	32
	piR-7107	4499	−151	86	32
	piR-1748	4542	−149	93	30
	piR-6746	4623	−142	85	31
	piR-3833	4645	−140	88	29
	piR-13641	4662	−142	93	28
	piR-13634	4663	−140	96	27
*EVI5*	piR-4815	3354	−142	84	31
	piR-10936	3378	−144	85	31
	piR-10886	3379	−142	84	30
	piR-10885	3379	−140	85	30
	piR-9994	3404	−153	91	30
	piR-9059	3404	−168	100	30
	piR-9036	3405	−142	88	29
	piR-3586	3407	−163	93	32
	piR-7244	3410	−153	96	29
	piR-5505	3441	−149	89	30
	piR-2344	3446	−151	93	30
	piR-1254	3517	−144	83	32
	piR-12623	3534	−144	88	30
	piR-4112	3543	−140	90	29
	piR-12340	3546	−140	90	28
	piR-17617	3550	−134	87	29
	piR-12397	3572	−142	92	28
	piR-12346	3570	−146	85	31
	piR-12399	3573	−155	97	28
	piR-5505	3577	−142	85	30
	piR-15405	3586	−155	94	30
	piR-14239	3586	−149	91	30
	piR-15404	3587	−149	93	29
	piR-14625	3598	−146	91	30
	piR-4815	4833	−146	86	31
	piR-4816	4833	−142	83	31
	piR-1254	4829	−157	90	32
	piR-12623	4847	−151	92	30
	piR-4112	4857	−140	90	29
	piR-12340	4859	−144	93	28
	piR-11421	4871	−144	86	30
	piR-11245	4872	−142	92	29
	piR-12346	4883	−144	84	31
	piR-12399	4886	−144	91	28
	piR-5505	4890	−140	84	30
	piR-15405	4899	−146	88	30
	piR-14239	4899	−140	86	30
	piR-15404	4900	−140	88	29
	piR-1248	5078	−142	91	29
	piR-10873	5078	−144	88	30
	piR-11596	5079	−142	88	30
	piR-10936	5078	−153	90	31
	piR-10886	5079	−157	93	30
	piR-10885	5079	−142	86	30
	piR-10873	5079	−153	94	30
	piR-10935	5079	−146	90	30
	piR-10934	5079	−144	91	30
	piR-10933	5079	−140	88	30
	piR-14091	5086	−132	89	28
	piR-9994	5104	−153	91	30
	piR-9059	5104	−161	96	30
	piR-9036	5105	−149	92	29
	piR-3586	5107	−168	95	32
	piR-7244	5110	−151	95	29
	piR-3587	5110	−142	91	29
	piR-1254	5215	−157	90	32
	piR-12623	5232	−144	88	30
	piR-17617	5248	−130	85	29
	piR-11421	5252	−140	84	30
	piR-12346	5268	−144	84	31
	piR-12399	5271	−142	89	28
	piR-15405	5284	−151	91	30
	piR-14239	5284	−144	88	30
	piR-15404	5285	−149	93	29
	piR-14625	5296	−149	92	30
	piR-9460	6836	−144	87	31
	piR-6746	6872	−142	85	31
	piR-5744	6872	−140	85	31
	piR-7107	6877	−146	83	32
	piR-7102	6878	−140	81	31
*IL12B*	piR-16315	1779	−144	86	30
	piR-9460	1783	−144	87	31
	piR-6746	1818	−146	87	31
	piR-15278	1822	−153	91	31
	piR-6105	1824	−144	84	31
	piR-7102	1824	−140	81	31
	piR-8561	1824	−140	81	32
	piR-16315	2076	−140	84	30
	piR-6236	2084	−144	89	30
	piR-5744	2115	−140	85	31
	piR-7107	2120	−149	84	32
	piR-7102	2121	−142	83	31
	piR-14883	2143	−146	86	31
	piR-14884	2145	−136	85	29
	piR-9460	2213	−149	90	31
	piR-13123	2215	−146	91	30
	piR-5363	2217	−140	90	29
	piR-8226	2233	−142	88	29
	piR-5744	2248	−146	88	31
	piR-6746	2248	−151	90	31
	piR-7107	2253	−146	83	32
	piR-15278	2252	−142	85	31
	piR-6105	2254	−144	84	31
	piR-7102	2254	−146	85	31
	piR-8561	2254	−151	88	32
	piR-6092	2254	−140	82	31
	piR-8561	2255	−149	86	32
	piR-225	2296	−142	91	28
	piR-14974	2304	−136	91	26
	piR-16075	2310	−138	92	28
*MLANA*	piR-1254	976	−151	87	32
	piR-4815	980	−140	82	31
	piR-13770	989	−142	93	28
	piR-13714	998	−144	97	27
	piR-1248	1005	−140	89	29
	piR-11596	1006	−140	87	30
	piR-10936	1005	−151	89	31
	piR-10886	1006	−155	91	30
	piR-10885	1006	−146	88	30
	piR-10873	1006	−151	92	30
	piR-10935	1006	−144	88	30
	piR-10934	1006	−142	89	30
	piR-10930	1006	−142	88	30
	piR-15670	1013	−136	86	28
	piR-9994	1031	−142	85	30
	piR-9059	1031	−157	94	30
	piR-3586	1034	−153	87	32
	piR-7244	1037	−142	89	29
	piR-5505	1068	−157	94	30
	piR-2344	1073	−142	88	30
	piR-15406	1077	−157	96	30
	piR-1254	1142	−155	89	32
	piR-12623	1159	−146	90	30
	piR-12340	1171	−144	93	28
	piR-17617	1175	−132	86	29
	piR-11421	1183	−142	85	30
	piR-12346	1195	−157	91	31
	piR-12399	1198	−149	93	28
	piR-15405	1211	−155	94	30
	piR-14239	1211	−149	91	30
	piR-15404	1212	−149	93	29
	piR-14625	1223	−144	89	30

## Data Availability

Data are contained within the present article.

## Supplementary Materials

The following supporting information can be downloaded at: https://www.mdpi.com/article/10.3390/nano13010022/s1, Table S1: List of MS target genes for miRNAs and piRNAs with an indication of the publication of the participation of the candidate gene in the development of MS; Table S2: Characteristics of miRNA interactions with 5′UTR mRNA of candidate MS genes; Table S3: Characteristics of miRNA interactions with CDS mRNA of candidate MS genes; Table S4: Characteristics of miRNA interactions with 3′UTR mRNA of candidate MS genes; Table S5: Characteristics of interaction between piRNA and 5’UTR mRNA of MS genes; Table S6: Characteristics of interaction between piRNA and CDS mRNA of MS genes.
