# Supplementary material for: piRNA and miRNA Can Suppress the Expression of Multiple Sclerosis Candidate Genes
Source: Nanomaterials (Basel). 2022 Dec 21;13(1):22. doi: 10.3390/nano13010022 (PMC9823834; doi:10.3390/nano13010022)
Supplement: Supplementary file 1 [file nanomaterials-13-00022-s001.zip › nanomaterials-2030593-supplementary.pdf]

Supplementary material to

piRNA and miRNA can Suppress the Expression of Multiple Sclerosis Candidate Genes

Table S1 List of MS target genes for miRNAs and piRNAs with an indication of the publication of the participation of the candidate gene in the development of MS.

Gene	PMID	Gene	PMID	Gene	PMID
ADAM17	16900751	*FOXP3*	33170805	*MLANA*	22534618
AHI1	29409597	*HLA-DRB1*	32646493	*MYC*	31912409
CD6	20430450	*IL2RA*	30352019	*SOX8*	23739915
CD86	32687946	*IL12B*	30554348	*TAGAP*	32312989
CD226	26359290	*IL-22RA2*	21041731	*TALDO1*	19401148
CLEC16A9	26359290	*IQGAP1*	27080863	*TBX21*	30886677
CYP27B1	24308945	*KIF21B*	20587413	*TNFRSF1A*	28927886
EOMES	29521285	*MAPK1*	34284104	*TRIP11*	33329728
EVI5	18401352	*MERTK*	33691116	*ZBTB46*	23739915
FCRL3	25862376	*MGAT5*	21115203	*ZMIZ1*	28275670
Note. Of the 30 candidate genes, nine genes were targets only for miRNAs (yellow), seven genes were targets only for piRNAs (green), and 14 genes were common targets for miRNAs and piRNAs (blue).					

Table S2 Characteristics of miRNA interactions with 5′UTR mRNA of candidate MS genes

Gene	miRNA	Start of site, nt	ΔG, kJ/mole	ΔG/ΔGm, %	Length, nt
CBLB5	miR-6865-3p	65	-106	91	21
CD6	ID01391.3p-miR	31	-115	90	23
CLEC16A9	miR-466	1368	-104	89	23
	ID00436.3p-miR	1380	-104	89	23
	miR-3529-3p	2240	-110	93	24
EOMES	ID00561.3p-miR	284	-119	92	21
	ID01759.3p-miR	289	-113	91	22
IL22RA2	b-miR-207-5p	30	-96	92	20
IL2RA	ID03161.3p-miR	76	-113	90	22
TBX21	miR-4783-5p	26	-117	92	21
ZBTB46	ID02080.3p-miR	91	-121	89	24

Table S3 Characteristics of miRNA interactions with CDS mRNA of candidate MS genes

Gene	miRNA	Start of site, nt	ΔG, kJ/mole	ΔG/ΔGm, %	Length, nt
CBLB5	ID01787.3p-miR	2895	-115	89	23
	miR-4459	2896	-115	90	22
CD6	ID00774.3p-miR	449	-121	90	23
CLEC16A9	b-miR-114-3p	3363	-110	90	22
	ID01734.5p-miR	3783	-119	95	22
CXCR5	miR-513a-5p	133	-91	96	18
	b-miR-1583-5p	381	-117	89	23
	b-miR-1584-5p	381	-117	89	23
FOXP3	b-miR-138-3p	1032	-102	91	21
IL12B	miR-6893-3p	803	-113	90	22
IQGAP1	miR-3681-3p	2147	-106	93	22
KIF21B	ID00777.3p-miR	2083	-113	90	23
	ID01759.3p-miR	2086	-113	91	22
	miR-3650	3370	-96	94	19
	ID02029.5p-miR	4536	-121	89	23
MERTK	ID02294.5p-miR	136	-129	88	24
	b-miR-2022-5p	1022	-108	93	22
	ID03188.5p-miR	1024	-104	89	23
SOX8	ID00555.3p-miR	273	-125	88	24
	ID01053.3p-miR	304	-127	90	24
	ID01667.3p-miR	320	-119	90	22
	ID02761.3p-miR	421	-136	91	24
	ID02773.3p-miR	1035	-115	93	20
	ID00296.3p-miR	1251	-138	88	25
	ID01377.3p-miR	1261	-117	92	20
	ID01879.5p-miR	1373	-123	91	22
	miR-6764-5p	1475	-110	91	22
TAGAP	b-miR-406-3p	772	-106	93	20
	miR-6824-5p	774	-117	93	22
TBX21	ID02256.3p-miR	498	-125	91	22
	ID01375.5p-miR	1007	-121	89	24
TNFRSF1A	miR-6756-5p	1361	-119	89	23
	ID01569.3p-miR	1708	-117	93	20
	ID02668.5p-miR	1709	-127	88	24
	ID01247.3p-miR	1712	-115	93	20
	b-miR-1883-5p	1713	-132	94	23
ZBTB46	b-miR-1556-5p	1615	-113	90	22
	ID01618.3p-miR	2058	-117	90	23
	ID01377.3p-miR	2130	-117	92	20
ZMIZ1	ID02333.3p-miR	3903	-117	90	22

Table S4 Characteristics of miRNA interactions with 3′UTR mRNA of candidate MS genes

Gene	miRNA	Start of site, nt	ΔG, kJ/mole	ΔG/ΔGm, %	Length, nt
CD6	ID01304.5p-miR	2389	-115	90	22
	miR-4507	2962	-113	96	20
CD86	miR-6784-3p	2295	-110	90	22
CXCR5	miR-920	1564	-102	92	20
	b-miR-34-5p	2043	-110	90	22
	miR-7843-5p	2279	-115	93	22
	miR-495-3p	2329	-100	90	22
	ID01970.3p-miR	2867	-113	90	23
	ID03324.3p-miR	2873	-115	90	22
	miR-6750-3p	3186	-106	91	21
	ID02379.3p-miR	3482	-119	89	24
	miR-6740-5p	3847	-106	91	22
CYP27B1	ID01558.3p-miR	2246	-115	89	23
FOXP3	ID00047.3p-miR	1715	-108	91	21
	miR-762	1867	-125	92	22
	miR-5088-5p	2428	-123	92	24
IL12B	miR-1303	1749	-106	91	22
	miR-619-5p	1800	-113	93	22
	miR-5585-3p	1807	-106	91	22
IL22RA2	ID02524.5p-miR	1808	-93	90	22
KIF21B	b-miR-2455-5p	5938	-115	90	22
	b-miR-1713-3p	6659	-125	88	24
	ID01713.5p-miR	6825	-115	92	20
	ID01562.3p-miR	6976	-117	90	22
	b-miR-594-5p	7261	-115	89	23
	ID00455.5p-miR	9440	-115	89	23
	miR-6881-5p	9454	-108	91	22
RPS6KB15	ID01661.3p-miR	2981	-104	91	21
SOX8	ID01262.3p-miR	1765	-113	93	21
	ID01911.5p-miR	2909	-123	89	23
	ID03153.3p-miR	2914	-121	90	22
	miR-6510-5p	2985	-113	91	22
TNFRSF1A	miR-6735-5p	2154	-121	88	25
ZBTB46	ID00648.5p-miR	2617	-125	92	22
	b-miR-1187-5p	3965	-117	90	23
	ID00382.5p-miR	4035	-113	93	20
	ID02460.5p-miR	4226	-117	90	22
	ID03288.5p-miR	4704	-117	90	23
ZMIZ1	miR-4433b-3p	4268	-110	91	21
	ID03374.3p-miR	4828	-108	91	21
	ID03475.5p-miR	4888	-110	90	22
	miR-6886-3p	5306	-106	91	21
	ID01450.5p-miR	5613	-113	91	22

Table S5 Characteristics of interaction between piRNA and 5'UTR mRNA of MS genes

Gene	miRNA	Start of site, nt	ΔG, kJ/mole	ΔG/ΔGm, %	Length, nt
MYC	piR-16771	469	-146	80	32
	piR-17232	663	-144	82	31
	piR-1177	672	-140	81	31
TAGAP	piR-7425	324	-142	85	32
TALDO1	piR-478	12	-140	85	28
TBX21	piR-6521	31	-140	80	31
TNFRSF1A	piR-4815	850	-144	85	31
	piR-10936	874	-149	87	31
	piR-10886	875	-159	94	30
	piR-10885	875	-144	87	30
	piR-1248	874	-144	92	29
	piR-10873	875	-149	91	30
	piR-10935	875	-142	87	30
	piR-10934	875	-140	88	30
TRIP11	piR-6009	280	-155	100	31
ZBTB46	piR-1254	37	-146	84	32
	piR-4815	41	-144	85	31
	piR-10933	67	-142	89	30
	piR-10934	67	-140	88	30
	piR-3586	99	-149	84	32
	piR-11421	114	-146	87	30

Table S6 Characteristics of interaction between piRNA and CDS mRNA of MS genes

Gene	miRNA	Start of site, nt	ΔG, kJ/mole	ΔG/ΔGm, %	Length, nt
CD6	piR-4828	370	-149	84	31
	piR-15162	590	-142	87	29
	piR-4985	1286	-140	80	32
EOMES	piR-478	778	-140	85	28
	piR-1468	914	-142	81	32
	piR-5938	1382	-140	80	32
	piR-5937	1383	-140	84	31
	piR-1344	1384	-140	80	32
FCRL3	piR-1811	1443	-146	87	30
	piR-3463	542	-149	85	31
HLA-DRB1	piR-3424	363	-151	85	32
	piR-14092	388	-144	81	31
KIF21B	piR-228	953	-142	83	31
	piR-8432	2470	-142	82	32
	piR-2846	4784	-140	81	32
SOX8	piR-9822	1345	-144	84	31
TAGAP	piR-13963	1321	-140	82	31
TALDO1	piR-13807	818	-140	82	31
TNFRSF1A	piR-14091	882	-132	89	28
	piR-15670	882	-142	91	28
	piR-9994	900	-144	86	30
	piR-9059	900	-159	95	30
	piR-9036	901	-140	87	29
	piR-3586	903	-155	88	32
	piR-7244	906	-144	91	29
	piR-5505	937	-157	94	30
	piR-2344	942	-149	92	30
	piR-15406	946	-144	88	30
ZMIZ1	piR-16650	858	-140	82	31
